# Correction: Morphometric evaluation of the anterior cranial fossa during the prenatal stage in humans and its clinical implications

**DOI:** 10.1371/journal.pone.0321125

**Published:** 2025-03-25

**Authors:** The PLOS One Staff

The reference list is incorrect. The correct reference list is:

1. Sandham A, Cheng L. Cranial base and cleft lip and palate. Angle Orthod. 1988 Apr:163–8.

2. Diewert VM. Development of human craniofacial morphology during the late embryonic and early fetal periods. Am J Orthod. 1985 Jul:88(1):64–76.

3. Ford HR. The growth of the foetal skull. J Anat Physiol. 1956;90:63–72.

4. Ricciardelli EJ. Embryology and anatomy of the cranial base. Clin Plast Surg. 1995 Jul;22(3):361–72. Review.

5. Friede H. Normal development and growth of the human neurocranium and cranial base. Scand J Plast Reconstr Surg. 1981;15:163–9.

6. Muller F, O’Rahilly R. The human chondrocranium at the end of the embryonic period, proper, with particular reference to the nervous system. Am J Anat. 1980;159:33–58.

7. Achiron R, Yagel S, Rotstein Z, Inbar O, Mashiach S, Lipitz S. Cerebral lateral ventricular asymmetry: is this a normal ultrasonographic finding in the fetal brain? Obstet Gynecol. 1997 Feb;89(2):233–7.

8. Kjaer I, Fischer-Hansen B. Human fetal pituitary gland in holoprosencephaly and anencephaly. J Craniofac Genet Dev Biol. 1995 Oct–Dec;15(4):222–9.

9. Stovner LJ, Bergan U, Nilsen G, Sjaastad O. Posterior cranial fossa dimensions in the Chiari I malformation: relation to pathogenesis and clinical presentation. Neuroradiology. 1993;35(2):113–8.

10. Nishikawa M, Sakamoto H, Hakuba A, Nakanishi N, Inoue Y. Pathogenesis of Chiari malformation: a morphometric study of the posterior cranial fossa. J Neurosurg. 1997 Jan;86(1):40–7.

11. Karagoz F, Izgi N, Kapijcijoglu Sencer S. Morphometric measurements of the cranium in patients with Chiari type I malformation and comparison with the normal population. Acta Neurochir (Wien). 2002 Feb;144(2):165–71; discussion 171.

12. Fields HW Jr, Metzner L, Garol JD, Kokich VG. The craniofacial skeleton in anencephalic human fetuses. I. Cranial floor. Teratology. 1978 Feb;17(1):57–65.

13. Nyberg DA. The fetal central nervous system. Semin Roentgenol. 1990 Oct;25(4):317–33.

14. Sgouros S, Natarajan K, Hockley AD, Goldin JH, Wake M. Skull base growth in craniosynostosis.

15. Kreiborg S, Jensen BL, Bjork A, Skieller V. Abnormalities of the cranial base in cleidocranial dysostosis. Am J Orthod. 1981 May;79(5):549–57.

16. Sadowska L, Wandokanty-Bochenska M, Domanasiewicz M, Pellar J, Wiraszka A, Kaplonska M. [Analysis of the effect of parental alcoholism on child development. III. Evaluation of the physical and psychomotor development in the first year of life of children of alcoholic parents]. Pediatr Pol. 1988 Feb;63(2):91–101.

17. Blaw ME, Woody RC. Teratology of the central nervous system. In: Rosenberg RN, editor. The clinical neurosciences- vol. 1. New York, London: Churchill Livingstone; 1983. p. I:1–I:32.

18. Carlson BM. Human embryology & developmental biology. New York: Mosby; 1999. str.450 p.

19. Dudek K, Kędzia W, Kędzia E, Kędzia A, Derkowski W. Mathematical modelling of the growth of human fetus anatomical structures. Anat Sci Int. 2017;92:521–9. doi: 10.1007/s12565-016-0353-y. Springer Japan.

20. Ellis PD. The essential guide to effect sizes: statistical power, meta-analysis, and the interpretation of research results. Cambridge University Press; 2010.

21. R Core Team. R Core Team R: a language and environment for statistical computing. Vienna, Austria: R foundation for statistical computing; 2021. Available from: https://www.R-project.org/

22. Domański J, Domagala Z, Simmons JE, Wanat M. Terra Incognita in anatomical museology - A literature review from the perspective of evidence-based care. Ann Anat. 2023 Jan;245:152013. Epub 2022 Oct 17. doi: 10.1016/j.aanat.2022.152013. PMID: 36257492.

23. Domański J, Janczura A, Wanat M, Wiglusz K, Grajzer M, Simmons JE, et al. Preservation fluids of heritage anatomical specimens - a challenge for modern science. Studies of the origin, composition and microbiological contamination of old museum collections. J Anat. 2023 Jul;243(1):148–66. Epub 2023 Apr 6. doi: 10.1111/joa.13854. PMID: 37024147; PMCID: PMC10273345.

24. Lee SK, Kim YS, Jo YA, Seo JW, Chi JG. Prenatal development of cranial base in normal Korean fetuses. Anat Rec. 1996 Dec;246(4):524–34.

25. Kędzia A, Rybaczuk M, Andrzejak R. 2002 Fractal dimensions of human brain cortex vessels during the fetal period. Med Sci Monit. 2002;8(3):46–51.

26. Kędzia A, Derkowski W. Modern methods of neuroanatomical and neurophysiological research. MethodsX. 2024. doi: 10.1016/j.mex.2024.102881. Available from: https://www.sciencedirect.com/science/article/pii/S2215016124003339

27. Ishimaru T. [Developmental studies on the palatine bone in the human skull. With special references to the development of its nasal surface]. Hokkaido Igaku Zasshi. 1984 May;59(3):299–311. Japanese.

28. Kjaer I. Prenatal human cranial development evaluated on coronal plane radiographs. J Craniofac Genet Dev Biol. 1990;10(4):339–51.

29. Bach-Petersen S, Solow B, Fischer-Hansen B, Kjaer I. Growth in the lateral part of the human skull during the second trimester. J Craniofac Genet Dev Biol. 1995 Oct–Dec;15(4):205–11.

30. Sherwood TF, Mooney MP, Sciote JJ, Smith TD, Cooper GM, Siegel MI. Cranial base growth and morphology in second-trimester normal human fetuses and fetuses with cleft lip. Cleft Palate Craniofac J. 2001 Nov;38(6):587–96.

31. Dimitriadis AS, Haritanti-Kouridou A, Antoniadis K, Ekonomou L. The human skull base angle during the second trimester of gestation. Neuroradiology. 1995 Jan;37(1):68-71.

32. Moon HJ, Kim HU, Lee JG, Chung IH, Yoon JH. Surgical anatomy of the anterior ethmoidal canal in ethmoid roof. Laryngoscope. 2001 May;111(5):900–4.

33. Jeffery N. A high-resolution MRI study of linear growth of the human fetal skull base. Neuroradiology. 2002 Apr;44(4):358–66. Epub 2002 Feb 16.

34. Sgouros S, Natarajan K, Hockley AD, Goldin JH, Wake M. Skull base growth in childhood. Pediatr Neurosurg. 1999 Nov;31(5):259–68.

35. Kapur E, Dilberovic F, Voljevica A, Talovic E. [Computer tomography study of the pterygopalatine fossa and its communications]. Med Arh. 2003;57(4):203–6. Croatian.

36. Schafer W. [Measurements on the grade of the human skull base and angle determinations]. Gegenbaurs Morphol Jahrb. 1975;121(1):1–25.

37. Paladino J, Gluncic V, Stern-Padovan R, Vinter I, Lukic IK, Marusic A. Cranial base kyphosis and the surface morphology of the anterior cranial fossa. Ann Anat. 2002 Jan;184(1):21–5.

38. Pouya F, Eftekhar-Vaghefi SH, Salehinejad P. Anthropometric analysis of cephalofacial dimensions in Kerman, Iran. Acta Medica Iranica. 2017:241–8.

39. Pouya F, Vaghefi SH, Raygan SP, Nejad FG. Cephalometry and determination of facial (Prosopic) index of Persian adolescents. Life Sci J. 2021;18(7):24–8.

40. Setianingsih H. The differences between male and female skulls in several anthropometric measurements; 2011.

41. Bilodi AKS, Gangadhar MR. A study on human skulls and its anthropological importance; 2014.

42. Nadel AS, Benacerraf BR. Lateral ventricular atrium: larger in male than female fetuses. Int J Gynaecol Obstet. 1995;51:123–6.

43. Glonek M, Kędzia A, Derkowski W. Prenatal assessment of ventriculomegaly: an anatomical study. Med Sci Monit. 2003;9(7):69–77.

44. Chong VF, Khoo JB, Fan YF. Imaging of the nasopharynx and skull base. Magn Reson Imaging Clin N Am. 2002 Nov;10(4):547–71, v. Review.

45. Iannetti G, Fadda MT, Indrizzi E, Gennaro P, Spuntarelli G. Scoliosis of the cranial base: radiological and mathematical analysis using finite elements system analysis (FESA) of a case. J Craniomaxillofac Surg. 2004 Aug;32(4):220–7.

46. Hattori T, Kobayashi H, Inoue S, Sakai N. Angiographically occult dural arteriovenous malformation in the anterior cranial fossa-case report. Neurol Med Chir (Tokyo). 1999 Apr;39(4):291–3.

47. Ciszek B, Ząbek M. [Anatomy of the middle cerebral artery and the angiographic image of its aneurysms]. Neurol Neurochir Pol. 1992;Suppl 1:67–72.

48. Kawaguchi T, Kawano T, Ooasa T, Ogasawara S, Uozumi Y. [The improvement in regional cerebral blood flow in the anterior cranial fossa which was affected by a dural arteriovenous fistula: case report]. No Shinkei Geka. 2003 Feb;31(2):209–14. Japanese.

49. Damante JH, Filho LI, Silva MA. Radiographic image of the hard palate and nasal fossa floor in panoramic radiography. Oral Surg Oral Med Oral Pathol Oral Radiol Endod. 1998 Apr;85(4):479–84.

50. Stazzone MM, Hubbard AM, Bilaniuk LT, Harty MP, Meyer JS, Zimmerman RA, et al. Ultrafast MR imaging of the normal posterior fossa in fetuses. AJR Am J Roentgenol. 2000 Sep;175(3):835–9.

51. Goldstein I, Makhoul IR, Tamir A, Rajamim BS, Nisman D. Ultrasonographic nomograms of the fetal fourth ventricle: additional tool for detecting abnormalities of the posterior fossa. J Ultrasound Med. 2002 Aug;21(8):849–56.

52. He J, Xia Y, Ying W, Liang F. [Study on clinical significance of fetal posterior fossa fluid]. honghua Fu Chan Ke Za Zhi. 2002 May;37(5):281–3. Chinese.

53. Glonek M, Kędzia A, Derkowski W. Prenatal assessment of ventriculomegaly: an anatomical study. Med Sci Monit. 2003;9(7):69–77.

54. Derkowski W, Kędzia A. Zastosowanie systemów przetwarzania obrazu Scion for Windows 98 i ELF do oceny rozwoju dołu przedniego czaszki człowieka w okresie prenatalnym. Materiały VIII Krajowa Konferencja Komputerowe Wspomaganie Badań Naukowych KOWBAN 2001. Wrocławskie Towarzystwo Naukowe; 2001. p. str.65–70.

55. Derkowski W, Kędzia A, Glonek M. Clinical anatomy of the human anterior cranial fossa during the prenatal period. Folia Morphol (Warsz). 2003;62(3):271–3.

56. Błaszczyk E. An evaluation of the size of the fourth ventricle in human foetuses. Folia Morphol (Warsz). 2004 May;63(2):241–3.

57. Kędzia W, Kędzia E, Kędzia A, Derkowski W. Anatomy of the falcine sinus during the prenatal period. Surg Radiol Anat. 2017;39:753–8. doi: 10.1007/s00276-016-1787-6. Springer Paris.

58. Grzonkowska M, Baumgart M, Badura M, Wiśniewski M, Szpinda M. Quantitative anatomy of the fused ossification center of the occipital squama in the human fetus. PLOS ONE. 2021;16(2):e0247601. https://doi.org/10.1371/journal.pone.0247601

59. Ziółko J. Zbiorniki metalowe na ciecze i gazy. Warszawa: Arkady; 1986.

60. Kędzia A, Derkowski W, Glonek M. Struktura a funkcja czaszki człowieka na podstawie badań metodami komputerowej analizy obrazu w okresie płodowym. Komputerowe wspomaganie badań naukowych tom 13. Wrocław Scientific Society; 2006. p. 241–6. ISBN 978-83-7374-043-3.

61. Alfieri A, Jho HD, Schettino R, Tschabitscher M. Endoscopic endonasal approach to the pterygopalatine fossa: anatomic study. Neurosurgery. 2003 Feb;52(2):374–78; discussion 378–80.

62. Dare AO, Balos LL, Grand W. Neural-dural transition at the medial anterior cranial base: an anatomical and histological study with clinical applications. J Neurosurg. 2003 Aug;99(2):362–5.

63. Meling TR, Due-Tonnessen BJ, Arctander K, Goodrich JT. [Transfacial neurosurgery in skull base tumors]. Tidsskr Nor Laegeforen. 2003 Feb 20;123(4):465–7. Norwegian.

64. Lorenz RR, Dean RL, Hurley DB, Chuang J, Citardi MJ. Endoscopic reconstruction of anterior and middle cranial fossa defects using acellular dermal allograft. Laryngoscope. 2003 Mar;113(3):496–501.

65. Ostrowski P, Bonczar M, Iwanaga J, Canon R, Dziedzic M, Kołodziejczyk B, et al. The cranio-orbital foramen: a meta-analysis with a review of the literature. Folia Morphol 2023;82(4):758–65. doi: 10.5603/FM.a2022.0086. Pubmed: 36178278

66. Captier G, Leboucq N, Bigorre M, Canovas F, Bonnel F, Bonnafe A, Montoya P. Plagiocephaly: morphometry of skull base asymmetry. Surg Radiol Anat. 2003 Sep 3

The publisher apologizes for the error.
