# Supplementary material for: Morphometric evaluation of the anterior cranial fossa during the prenatal stage in humans and its clinical implications
Source: PLoS One. 2024 Dec 27;19(12):e0309184. doi: 10.1371/journal.pone.0309184 (PMC11676864; doi:10.1371/journal.pone.0309184)
Supplement: S1 Appendix — (DOCX) [file pone.0309184.s001.docx]

**Appendix.**

Descriptive statistics of the distances between the characteristic points of the skull of 77 fetuses and the results of comparisons:

| Parameters | All | Male | Female | M *vs* F |
| --- | --- | --- | --- | --- |
| *FS-SA* (mm) |  |  |  | *Z* = -1.065  *p* = 0.287 |
| *M* (*SD*) | 13.6 (3.2) | 13.1 (2.7) | 14.5 (3.7) |  |
| *Me* [*Q*1; *Q*3] | 13.4 [12.0; 15.4] | 13.2 [12.0; 14.7] | 14.0 [11.8; 16.5] |  |
| *Min* - *Max* | 4.7 - 23.1 | 4.7 - 18.4 | 9.6 - 23.1 |  |
| *FS-CS* (mm) |  |  |  | *Z* = -0.507  *p* = 0.612 |
| *M* (*SD*) | 5.3 (2.1) | 5.0 (1.9) | 5.8 (2.3) |  |
| *Me* [*Q*1; *Q*3] | 5.0 [4.3; 5.6] | 5.0 [4.0; 5.6] | 5.0 [4.4; 5.9] |  |
| *Min* - *Max* | 1.1 - 13.1 | 1.1 - 13.1 | 3.5 - 12.9 |  |
| *CS-SA* (mm) |  |  |  | *Z* = -0.924  *p* = 0.355 |
| *M* (*SD*) | 8.4 (3.2) | 8.0 (2.7) | 9.0 (3.8) |  |
| *Me* [*Q*1; *Q*3] | 8.2 [5.6; 10.5] | 8.2 [5.8; 9.7] | 8.4 [5.3; 10.9] |  |
| *Min* - *Max* | 3.6 - 19.5 | 3.6 - 14.5 | 4.7 - 19.5 |  |
| *SA-SP* (mm) |  |  |  | *Z* = 0.068  *p* = 0.946 |
| *M* (*SD*) | 9.6 (2.5) | 9.5 (2.5) | 9.7 (2.4) |  |
| *Me* [*Q*1; *Q*3] | 9.3 [8.0; 11.0] | 9.5 [8.0; 11.4] | 9.0 [8.1; 10.1] |  |
| *Min* - *Max* | 3.1 - 15.1 | 3.1 - 14.5 | 6.4 - 15.1 |  |
| *FS-SP* (mm) |  |  |  | *Z* = -0.616  *p* = 0.538 |
| *M* (*SD*) | 22.7 (5.2) | 22.2 (5.2) | 23.5 (5.2) |  |
| *Me* [*Q*1; *Q*3] | 23.1 [18.3; 26.8] | 23.0 [18.3; 26.8] | 23.9 [18.2; 26.4] |  |
| *Min* - *Max* | 7.2 - 35.6 | 7.2 - 31.2 | 16.0 - 35.6 |  |
| *FS-X* (mm) |  |  |  | *Z* = -0.825  *p* = 0.409 |
| *M* (*SD*) | 51.5 (9.3) | 50.7 (9.2) | 52.8 (9.5) |  |
| *Me* [*Q*1; *Q*3] | 51.2 [44.0; 58.1] | 51.1 [43.7; 57.9] | 52.0 [44.5; 59.9] |  |
| *Min* - *Max* | 32.3 - 74.9 | 32.3 - 68.7 | 35.2 - 74.9 |  |
| *X-MP* (mm) |  |  |  | *Z* = -0.298  *p* = 0.766 |
| *M* (*SD*) | 26.2 (6.0) | 26.2 (6.0) | 26.1 (6.0) |  |
| *Me* [*Q*1; *Q*3] | 25.6 [22.8; 28.0] | 25.6 [22.8; 27.2] | 25.6 [22.1; 29.1] |  |
| *Min* - *Max* | 15.4 - 47.8 | 15.4 - 47.8 | 17.2 - 41.4 |  |
| *FS-MP* (mm) |  |  |  | *Z* = -0.862  *p* = 0.389 |
| *M* (*SD*) | 39.5 (7.9) | 38.7 (8.0) | 40.8 (7.5) |  |
| *Me* [*Q*1; *Q*3] | 39.7 [35.1; 45.2] | 39.6 [32.8; 44.5] | 40.5 [35.3; 46.6] |  |
| *Min* - *Max* | 16.8 - 53.1 | 16.8 - 51.9 | 27.1 - 53.1 |  |
| *FS-MA* (mm) |  |  |  | *Z* = -1.045  *p* = 0.296 |
| *M* (*SD*) | 35.2 (7.2) | 34.3 (7.2) | 36.6 (7.0) |  |
| *Me* [*Q*1; *Q*3] | 35.3 [30.1; 41.1] | 35.0 [29.2; 40.1] | 36.0 [30.8; 42.2] |  |
| *Min* - *Max* | 14.8 - 49.5 | 14.8 - 46.4 | 25.8 - 49.5 |  |
| *MA-MP* (mm) |  |  |  | *Z* = -0.313  *p* = 0.754 |
| *M* (*SD*) | 4.5 (0.9) | 4.5 (0.9) | 4.6 (1.0) |  |
| *Me* [*Q*1; *Q*3] | 4.6 [3.8; 4.9] | 4.6 [3.8; 4.9] | 4.4 [3.7; 5.2] |  |
| *Min* - *Max* | 2.5 - 7.6 | 2.5 - 7.6 | 3.0 - 6.7 |  |
| *FS-Y* (mm) |  |  |  | *Z* = -0.360  *p* = 0.719 |
| *M* (*SD*) | 50.3 (16.0) | 50.1 (18.1) | 50.5 (12.3) |  |
| *Me* [*Q*1; *Q*3] | 48.3 [40.2; 58.1] | 47.3 [40.2; 58.4] | 49.8 [38.3; 58.0] |  |
| *Min* - *Max* | 19.6 - 113.5 | 19.6 - 113.5 | 34.2 - 79.2 |  |
| *X-Y* (mm) |  |  |  | *Z* = 0.538  *p* = 0.591 |
| *M* (*SD*) | 64.2 (14.4) | 65.3 (15.7) | 62.5 (12.3) |  |
| *Me* [*Q*1; *Q*3] | 63.0 [54.8; 71.9] | 63.0 [55.0; 77.0] | 63.2 [54.8; 67.6] |  |
| *Min* - *Max* | 33.6 - 111.5 | 33.6 - 111.5 | 35.8 - 92.5 |  |

| Parameters | All | Male | Female | M *vs* F |
| --- | --- | --- | --- | --- |
| *S-F* (mm), distance from the pituitary gland center to nasion | | | | *Z* = -0.005  *p* = 0.996 |
| *M* (*SD*) | 26.7 (5.3) | 26.4 (5.7) | 27.2 (4.6) |  |
| *Me* [*Q*1; *Q*3] | 28.0 [23.5; 30.3] | 28.0 [23.5; 30.5] | 27.8 [23.2; 30.0] |  |
| *Min* - *Max* | 8.8 - 36.4 | 8.8 - 33.7 | 16.9 - 36.4 |  |
| *S-ZL* (mm), distance from the pituitary gland center to the left zygomatic bone | | | | *Z* = 0.245  *p* = 0.806 |
| *M* (*SD*) | 21.3 (4.3) | 21.1 (4.8) | 21.5 (3.6) |  |
| *Me* [*Q*1; *Q*3] | 22.4 [19.2; 24.0] | 22.6 [19.2; 24.0] | 21.9 [18.6; 24.2] |  |
| *Min* - *Max* | 6.5 - 27.8 | 6.5 - 27.7 | 13.0 - 27.8 |  |
| *S-ZR* (mm), distance from the pituitary gland center to the right zygomatic bone | | | | *Z* = 1.018  *p* = 0.308 |
| *M* (*SD*) | 22.0 (4.3) | 22.1 (4.9) | 21.8 (3.2) |  |
| *Me* [*Q*1; *Q*3] | 22.9 [19.9; 24.6] | 23.4 [19.9; 25.0] | 22.4 [19.5; 24.5] |  |
| *Min* - *Max* | 7.2 - 28.9 | 7.2 - 28.9 | 13.2 - 26.8 |  |
| *S-ZL vs S-ZR* | *Z* = 3.365  ***p = 0.001*** | *Z* = 3.889  ***p < 0.001*** | *Z* = 0.930  *p* = 0.352 | × |
| *F-ZL* (mm), distance between the nasion point and the left zygomatic bone | | | | *Z* = 1.572  *p* = 0.116 |
| *M* (*SD*) | 27.6 (5.3) | 28.1 (5.9) | 26.9 (4.0) |  |
| *Me* [*Q*1; *Q*3] | 28.6 [25.1; 31.1] | 29.0 [25.5; 31.7] | 26.1 [24.3; 30.2] |  |
| *Min* - *Max* | 9.4 - 36.7 | 9.4 - 36.7 | 16.9 - 34.4 |  |
| *F-ZR* (mm), distance between the nasion point and the right zygomatic bone | | | | *Z* = 1.316  *p* = 0.188 |
| *M* (*SD*) | 28.8 (5.6) | 29.2 (6.3) | 28.1 (4.5) |  |
| *Me* [*Q*1; *Q*3] | 29.9 [25.6; 32.2] | 30.7 [26.7; 32.6] | 28.3 [25.3; 31.5] |  |
| *Min* - *Max* | 9.6 - 38.5 | 9.6 - 38.5 | 17.2 - 37.1 |  |
| *F-ZL vs F-ZR* | *Z* = 5.862  ***p = 0.001*** | *Z* = 4.646  ***p = 0.001*** | *Z* = 3.517  ***p = 0.001*** | × |
| *S-PL* (mm), distance between the S point and the left ear cartilage | | | | *Z* = 1.123  *p* = 0.261 |
| *M* (*SD*) | 27.4 (4.6) | 27.6 (5.1) | 27.1 (3.8) |  |
| *Me* [*Q*1; *Q*3] | 27.4 [25.3; 30.9] | 28.3 [26.0; 31.0] | 27.0 [24.3; 30.5] |  |
| *Min* - *Max* | 7.8 - 34.0 | 7.8 - 33.9 | 18.6 - 34.0 |  |
| *S-PR* (mm), distance between the S point and the right ear cartilage | | | | *Z* = 1.076  *p* = 0.282 |
| *M* (*SD*) | 27.1 (4.5) | 27.3 (5.2) | 27.0 (3.3) |  |
| *Me* [*Q*1; *Q*3] | 28.0 [25.6; 30.0] | 28.2 [26.1; 30.6] | 27.4 [23.8; 29.6] |  |
| *Min* - *Max* | 8.1 - 33.6 | 8.1 - 33.6 | 18.5 - 33.3 |  |
| *S-PL vs S-PR* | *Z* = 1.963  *p* = 0.053 | *Z* = 1.726  *p* = 0.064 | *Z* = 0.946  *p* = 0.344 | × |
| *ZL-PL* (mm), distance between the ossification point ZL and the point PL | | | | *Z* = -0.930  *p* = 0.353 |
| *M* (*SD*) | 25.3 (5.2) | 24.6 (5.3) | 26.2 (4.9) |  |
| *Me* [*Q*1; *Q*3] | 26.4 [22.4; 28.1] | 26.2 [22.4; 27.9] | 26.9 [21.8; 29.2] |  |
| *Min* - *Max* | 6.0 - 35.5 | 6.0 - 34.1 | 17.1 - 35.5 |  |
| *ZR-PR* (mm), distance between the ossification point ZR and the point PR | | | | *Z* = -0.073  *p* = 0.942 |
| *M* (*SD*) | 24.6 (5.0) | 24.2 (5.4) | 25.2 (4.3) |  |
| *Me* [*Q*1; *Q*3] | 25.2 [22.3; 27.7] | 25.5 [22.0; 27.7] | 24.3 [22.4; 27.7] |  |
| *Min* - *Max* | 5.3 - 33.6 | 5.3 - 32.5 | 16.8 - 33.6 |  |
| *ZL-PL vs ZR-PR* | *Z* = 3.793  ***p < 0.001*** | *Z* = 2.783  ***p = 0.005*** | *Z* = 2.612  ***p = 0.009*** | × |

| Parameters | All | Male | Female | M *vs* F |
| --- | --- | --- | --- | --- |
| *PL-PR* (mm) |  |  |  | *Z* = 1.536  *p* = 0.125 |
| *M* (*SD*) | 41.7 (8.1) | 42.0 (9.4) | 41.2 (5.5) |  |
| *Me* [*Q*1; *Q*3] | 43.1 [38.6; 46.7] | 43.7 [39.8; 47.6] | 42.0 [37.3; 44.2] |  |
| *Min* - *Max* | 13.5 - 55.6 | 13.5 - 55.6 | 25.6 - 52.4 |  |
| *CL1-CL2* (mm) |  |  |  | *Z* = 0.475  *p* = 0.635 |
| *M* (*SD*) | 10.3 (2.5) | 10.3 (2.7) | 10.4 (2.3) |  |
| *Me* [*Q*1; *Q*3] | 10.6 [8.9; 12.1] | 10.8 [9.3; 12.1] | 10.3 [8.7; 12.0] |  |
| *Min* - *Max* | 2.9 - 14.8 | 2.9 - 14.8 | 5.5 - 14.8 |  |
| *CR1-CR2* (mm) |  |  |  | *Z* = 0.653  *p* = 0.514 |
| *M* (*SD*) | 10.5 (2.5) | 10.5 (2.7) | 10.5 (2.2) |  |
| *Me* [*Q*1; *Q*3] | 11.0 [9.2; 12.3] | 11.1 [9.3; 12.4] | 10.6 [8.8; 12.2] |  |
| *Min* - *Max* | 3.1 - 15.3 | 3.1 - 14.0 | 5.8 - 15.3 |  |
| *CL1-CL2 vs*  *CR1-CR2* | *Z* = 3.143  ***p = 0.002*** | *Z* = 3.338  ***p = 0.001*** | *Z* = 0.854  *p* = 0.393 | × |
| *CL3-CL4* (mm) |  |  |  | *Z* = -0.548  *p* = 0.583 |
| *M* (*SD*) | 3.0 (0.8) | 2.9 (0.8) | 3.1 (0.9) |  |
| *Me* [*Q*1; *Q*3] | 3.0 [2.6; 3.4] | 3.0 [2.5; 3.3] | 3.0 [2.6; 3.6] |  |
| *Min* - *Max* | 0.7 - 5.3 | 0.7 - 4.4 | 0.9 - 5.3 |  |
| *CR3-CR4* (mm) |  |  |  | *Z* = -2.068  ***p = 0.039*** |
| *M* (*SD*) | 2.8 (1.0) | 2.6 (0.8) | 3.2 (1.2) |  |
| *Me* [*Q*1; *Q*3] | 2.8 [2.2; 3.3] | 2.6 [2.2; 3.1] | 3.1 [2.4; 3.6] |  |
| *Min* - *Max* | 0.9 - 6.3 | 0.9 - 4.3 | 1.2 - 6.3 |  |
| *CL3-CL4 vs*  *CR3-CR4* | *Z* = 1.742  *p* = 0.081 | *Z* = 2.799  ***p = 0.005*** | *Z* = 0.781  *p* = 0.435 | × |
| *B-AL* (mm) |  |  |  | *Z* = 0.611  *p* = 0.541 |
| *M* (*SD*) | 20.9 (3.6) | 20.8 (4.2) | 21.1 (2.3) |  |
| *Me* [*Q*1; *Q*3] | 21.5 [19.4; 23.1] | 21.8 [19.0; 23.3] | 21.2 [19.7; 22.7] |  |
| *Min* - *Max* | 7.6 - 26.3 | 7.6 - 26.3 | 15.4 - 25.0 |  |
| *B-AR* (mm) |  |  |  | *Z* = 1.358  *p* = 0.174 |
| *M* (*SD*) | 20.8 (3.4) | 20.9 (3.8) | 20.6 (2.5) |  |
| *Me* [*Q*1; *Q*3] | 21.7 [19.4; 23.1] | 22.3 [20.0; 23.4] | 20.8 [19.1; 22.5] |  |
| *Min* - *Max* | 6.6 - 25.9 | 6.6 - 25.9 | 13.6 - 24.1 |  |
| *B-AL vs B-AR* | *Z* = 1.333  *p* = 0.182 | *Z* = 0.011  *p* = 0.991 | *Z* = 2.088  ***p = 0.037*** | × |
| *O-AL* (mm) |  |  |  | *Z* = -0.136  *p* = 0.892 |
| *M* (*SD*) | 24.9 (4.5) | 24.6 (5.0) | 25.4 (3.6) |  |
| *Me* [*Q*1; *Q*3] | 25.8 [22.7; 27.9] | 25.9 [22.2; 27.5] | 25.7 [23.5; 28.0] |  |
| *Min* - *Max* | 7.8 - 32.6 | 7.8 - 30.6 | 16.7 - 32.6 |  |
| *O-AR* (mm) |  |  |  | *Z* = -0.136  *p* = 0.892 |
| *M* (*SD*) | 24.9 (4.3) | 24.7 (4.7) | 25.3 (3.7) |  |
| *Me* [*Q*1; *Q*3] | 25.6 [23.7; 27.4] | 25.7 [23.2; 27.6] | 25.6 [23.8; 27.4] |  |
| *Min* - *Max* | 6.7 - 33.8 | 6.7 - 33.3 | 17.4 - 33.8 |  |
| *O-AL vs O-AR* | *Z* = 0.843  *p* = 0.399 | *Z* = 0.587  *p* = 0.557 | *Z* = 0.463  *p* = 0.644 | × |
| *AL-AR* (mm) |  |  |  | *Z* = 0.303  *p* = 0.762 |
| *M* (*SD*) | 38.2 (5.6) | 37.9 (6.5) | 38.6 (3.9) |  |
| *Me* [*Q*1; *Q*3] | 39.5 [35.9; 41.9] | 39.7 [35.8; 42.1] | 38.5 [36.4; 41.7] |  |
| *Min* - *Max* | 13.7 - 45.9 | 13.7 - 45.9 | 28.1 - 45.2 |  |

Proportions of the depths and angles of the cranial fossae in male and female fetuses and results of statistical significance tests:

| Parameters | All | Male | Female | M *vs* F |
| --- | --- | --- | --- | --- |
| *hm/hp* (%) |  |  |  | *Z* = -0.360  *p* = 0.719 |
| *M* (*SD*) | 23.4 (7.7) | 23.0 (7.4) | 24.0 (8.4) |  |
| *Me* [*Q*1; *Q*3] | 23.6 [17.6; 27.8] | 24.1 [17.6; 27.5] | 23.6 [17.6; 29.4] |  |
| *Min* - *Max* | 0.0 - 40.0 | 0.0 - 40.0 | 0.0 - 40.0 |  |
| *hc/hp* (%) |  |  |  | *Z* = -2.920  *p* = 0.112 |
| *M* (*SD*) | 9.3 (4.5) | 8.3 (4.4) | 10.9 (4.1) |  |
| *Me* [*Q*1; *Q*3] | 8.3 [6.3; 11.8] | 8.2 [5.6; 10.3] | 10.5 [8.2; 13.0] |  |
| *Min* - *Max* | 0.0 - 25.0 | 0.0 - 22.2 | 4.2 - 25.0 |  |
| *hc/hm* (%) |  |  |  | *Z* = -2.448  *p* = 0.070 |
| *M* (*SD*) | 41.3 (21.9) | 37.7 (23.0) | 47.1 (19.0) |  |
| *Me* [*Q*1; *Q*3] | 40.0 [25.0; 50.0] | 34.6 [20.0; 47.8] | 43.3 [35.4; 50.0] |  |
| *Min* - *Max* | 0.0 - 100.0 | 0.0 - 100.0 | 16.7 - 100.0 |  |
| *a/p* (-) |  |  |  | *Z* = 2.011  *p* = 0.132 |
| *M* (*SD*) | 1.43 (0.29) | 1.48 (0.34) | 47.1 (19.0) |  |
| *Me* [*Q*1; *Q*3] | 1.40 [1.26; 1.51] | 1.43 [1.34; 1.54] | 43.3 [35.4; 50.0] |  |
| *Min* - *Max* | 1.08 - 2.96 | 1.13 - 2.96 | 16.7 - 100.0 |  |
| *a/m* (-) |  |  |  | *Z* = 2.846  *p* = 0.112 |
| *M* (*SD*) | 1.21 (0.28) | 37.7 (23.0) | 47.1 (19.0) |  |
| *Me* [*Q*1; *Q*3] | 1.19 [1.02; 1.39] | 34.6 [20.0; 47.8] | 43.3 [35.4; 50.0] |  |
| *Min* - *Max* | 0.77 - 2.50 | 0.0 - 100.0 | 16.7 - 100.0 |  |
| *p/m* (-) |  |  |  | *Z* = 1.165  *p* = 0.244 |
| *M* (*SD*) | 0.87 (0.21) | 37.7 (23.0) | 47.1 (19.0) |  |
| *Me* [*Q*1; *Q*3] | 0.86 [0.77; 0.95] | 34.6 [20.0; 47.8] | 43.3 [35.4; 50.0] |  |
| *Min* - *Max* | 0.36 - 1.83 | 0.0 - 100.0 | 16.7 - 100.0 |  |
